# Hyperbaric oxygen as an adjunctive therapy in treatment of malignancies, including brain tumours

**DOI:** 10.1007/s12032-016-0814-0

**Published:** 2016-08-02

**Authors:** Katarzyna Stępień, Robert P. Ostrowski, Ewa Matyja

**Affiliations:** Department of Experimental and Clinical Neuropathology, Mossakowski Medical Research Centre, Polish Academy of Sciences, 5 Pawińskiego Str., 02-106 Warsaw, Poland

**Keywords:** Hyperbaric oxygen therapy, Glioblastoma, Cancer, Hypoxia, Radiation therapy, Chemotherapy

## Abstract

Hyperbaric oxygen (HBO) therapy is widely used as an adjunctive treatment for various pathological states, predominantly related to hypoxic and/or ischaemic conditions. It also holds promise as an approach to overcoming the problem of oxygen deficiency in the poorly oxygenated regions of the neoplastic tissue. Occurrence of local hypoxia within the central areas of solid tumours is one of the major issues contributing to ineffective medical treatment. However, in anti-cancer therapy, HBO alone gives a limited curative effect and is typically not applied by itself. More often, HBO is used as an adjuvant treatment along with other therapeutic modalities, such as radio- and chemotherapy. This review outlines the existing data regarding the medical use of HBO in cancer treatment, with a particular focus on the use of HBO in the treatment of brain tumours. We conclude that the administration of HBO can provide many clinical benefits in the treatment of tumours, including management of highly malignant gliomas. Applied immediately before irradiation, it is safe and well tolerated by patients, causing rare and limited side effects. The results obtained with a combination of HBO/radiotherapy protocol proved to be especially favourable compared to radiation treatment alone. HBO can also increase the cytostatic effect of certain drugs, which may render standard chemotherapy more effective. The currently available data support the legitimacy of conducting further research on the use of HBO in the treatment of malignancies.

## Introduction

Hyperbaric oxygen (HBO) therapy is the use of oxygen under elevated atmospheric pressure, that is, at a pressure higher than the pressure found on the surface of the earth at sea level, which is defined to be 1 atm [1]. Currently, hyperbaric oxygenation is widely used as an adjunctive treatment for various pathological states, predominantly related to hypoxic and/or ischaemic conditions. The standard protocol for hyperbaric oxygen therapy (HBOT) specifies that patients breathe pure oxygen (~100 %) under pressure between 1.5 and 2.5 atmospheres absolute (ATA), which is defined as the sum of the atmospheric pressure and the gauge pressure inside the hyperbaric chamber.

Oxygen is transported in blood to tissues by two different, well-known mechanisms: in complex with haemoglobin in red blood cells (RBCs) and dissolved in blood plasma. Under normal atmospheric conditions, almost 97 % of the available haemoglobin is saturated with oxygen. In contrast, plasma typically contains only 0.32 % dissolved oxygen [2]. Thus, the administration of HBO does not have a large effect on oxygen delivery *via* red blood cells, but may improve haemoglobin-independent transport. HBO has additional beneficial effects, refining the elasticity of RBCs and reducing platelet aggregation, which are especially important when the underlying cause of the tissue hypoxia is cardiovascular in origin [1]. According to Henry’s law, if the partial pressure of oxygen (pO_2_) rises, the oxygen content in tissues will also increase [3]. Under higher pO_2_, the distance that oxygen diffuses is increased. This phenomenon, along with the property that oxygen that is dissolved in fluid can reach areas that are inaccessible to RBCs, allows one to imagine that breathing hyperbaric 100 % oxygen may enable more efficient oxygenation of tissues with defective vascularization. HBOT increases the amount of dissolved oxygen in the blood and therefore enhances tissue oxygenation. HBOT has other beneficial biochemical and cellular effects, including reduction in oedema, constriction of blood vessels, activation of phagocytosis, neovascularization, and stimulation of collagen production by fibroblasts. Consequently, HBO finds application in the management of various pathological states [3, 4]. Its beneficial role has been well established in treatment of ischaemia and reperfusion injury [5–7]. HBO has also become widely used as an adjunctive treatment for a variety of challenging medical conditions, including arterial gas embolism [8, 9], carbon monoxide poisoning [10–12], delayed radiation injury [13–16], decompression sickness [17], problematic wounds [18], bone fractures [19], and liver dysfunction [20]. HBO appears to be generally safe for patients, as its side effects are rare and oxygen toxicity appears primarily when it is used at very high doses and for a longer duration than is recommended [4].

This review outlines the existing data regarding the medical use of HBO in cancer treatment, with a particular focus on the use of HBOT in the treatment of brain tumours and for prevention of late radiation injury in brain tissue.

## Hyperbaric oxygen therapy in cancer treatment

One of the major issues contributing to ineffective medical treatment of cancer is the occurrence of local hypoxia within the central tumour areas. The application of HBOT may help overcome the problem of oxygen deficiency in the poorly oxygenated regions of the neoplastic tissue. To date, several reviews on the use of HBO in cancer treatment have been published [2, 21, 22]. The effect of this therapy on malignancy has been unclear for a long time. The first and foremost question was whether HBO inhibits tumour growth or perhaps even enhances growth and the ability to form metastases. This fear grew from the observation in previous research studies that HBOT has a confirmed stimulatory effect on the proliferation of fibroblasts and epithelial cells in wound healing [3, 23]. Initially, the impact of hyperbaric oxygen on cancer cells was not clearly defined, and the results of early studies were controversial. McMillan et al. [24] concluded that HBO suppresses the development of oral carcinoma during its induction phase, whereas HBO might promote tumour growth during the proliferation phase. Recently, the similar results were obtained by Doguchi et al. [25] who claimed that hyperbaric oxygenation enhances tumour cells proliferation and therefore promotes chemically induced skin carcinogenesis. Braks et al. [26] conducted in vivo study on head and neck squamous cell carcinoma in mouse model, which indicated that HBO alone can enhance tumour growth, hypoxia, and vascular permeability of non-irradiated tumours. No changes in time survival, metastasis, and malignancy of cancer were observed. However, it should be emphasized that in the presented studies time and frequency of oxygenation were greater than in most experimentally used protocols. Nevertheless, the current state of knowledge almost unequivocally states that HBO is not only unconducive to further cancer development, but even might reduce the main tumour mass [27, 28]. It has been documented that HBO contributes to reduced tumour hypoxia by raising the oxygen concentration in the blood plasma. Brizel et al. [29] performed in vivo studies on rats with implanted mammary adenocarcinoma. Animals divided into five groups were treated with inhaled normobaric or hyperbaric (3 ATA) oxygen or with normobaric or hyperbaric (3 ATA) carbogen (a gas mixture consisting of 95 % O_2_ and 5 % CO_2_). There was no significant improvement in tumour oxygenation in groups exposed to either normobaric oxygen or carbogen. However, positive results were observed in the groups treated with high-pressure gases. The latest analogical study was conducted by Thews and Vaupel [30]. They tested an influence of normo- and hyperbaric hyperoxygenation (pure oxygen or carbogen) on local hypoxic regions occurrence and spatial pO_2_ distribution using DS-sarcoma cell line injected subcutaneously in rats. While in standard conditions (1 atm) hyperoxia only slightly enhanced tumour oxygenation in comparison with normal air, under pressure of 2 atm median pO_2_ was fivefold higher. After hyperbaric oxygen as well as hyperbaric carbogen administration, the spatial pO_2_ distribution profiles showed almost absolute elimination of hypoxic regions, even in the large tumours. However, HBO alone gives a limited curative effect and is typically not applied by itself. More often, HBO is used as an adjuvant treatment along with other therapeutic modalities, such as radio-, chemo-, and photodynamic therapy. Recently, Poff et al. [31] proposed also non-toxic, metabolic anti-cancer therapy based on ketonic diet, ketone supplementation, and hyperbaric oxygen combination. When used during surgical procedures, HBO helps to speed the convalescence and healing of wounds, and it minimizes surgical pain [32].

### Hyperbaric oxygen as an radiotherapy adjuvant

Radiotherapy (RT) utilizes the so-called classical oxygen effect in tumour treatment. Upon exposure to radiation, water molecules undergo radiolysis to form unstable hydrogen and hydroxyl radicals. Hydrogen radicals react with molecular oxygen, yielding unstable perhydroxyl radicals and hydrogen peroxide, which cause serious DNA strand damage and consequently lead to cell death [33]. Thus, radiation treatment gives an optimal therapeutic result in well-oxygenated tumour tissue. It was observed that mice breathing pure 1 atm oxygen required a one-third smaller dose of X-rays than mice that were breathing air to achieve similar cancer regression [34]. HBO might play two possible roles when combined with RT: it may act as a radiosensitizer, which enhances the effect of radiation, or it may act as a therapeutic agent, reducing delayed radiation injury [13–16]. A combination of HBO and radiotherapy reduces tumour growth and improves local tumour control, resulting in increased survival time [21, 35]. Watson et al. [36] conducted clinical trial on 320 cervical carcinoma patients to investigate the influence of radiation in combination with HBO. Both local tumour control and patient survival in the HBO group were significantly better than in the control normobaric group. HBO had additional impressive positive effects: oxygen therapy abolished negative side effects of RT such as the accompanying postradiation injury of normal tissue. Similar results were obtained by Cade and McEwen, who enrolled 505 patients with various types of cancer into a clinical trial investigating the impact of HBO with radiotherapy [37]. Low doses of radiation provided a weak therapeutic effect; however, administration of a maximum dose along with high-pressure oxygen improved the survival of the patients with cervical or bronchial carcinoma in comparison with patients who were irradiated in normal air. However, the combined therapy did not affect patients with bladder tumours [37]. Also, Henk et al. [38] obtained similar negative results in a clinical trial of head and neck cancer treatment in which HBO was used as a radiosensitizer. In this trial, the survival time was similar regardless of whether the patients were breathing oxygen under elevated pressure or normobaric air; however, local control of the tumour was improved in the HBO-treated group. In further studies, Henk et al. [39] observed a significant improvement in patient survival (60 vs. 30 %) and tumour local control (63 vs. 30 %) with radiation treatment in HBO or air. Some other research groups, who conducted similar independent experiments, noted a high prevalence of severe late complications [40–43]. Other recent reports suggest that HBO treatment of patients with cancers of the head and neck improves not only local tumour control but also the overall outcome and that the side effects observed previously were caused by excessive radiation doses per fraction [35, 44].

### Hyperbaric oxygen as a chemotherapy adjuvant

The mechanism of action of certain cytostatic drugs is based on the production of reactive oxygen species (ROS). ROS are formed as a result of oxidative stress in eukaryotic cells, triggered by either insufficient or excessive levels of oxygen in tissue, by radiation, by toxins, or by other adverse factors [33]. ROS cause cellular damage *via* oxidation, and this has been implicated in the pathogenesis of many diseases. Increased production of ROS results in DNA strand damage, apoptosis, and cell death. However, the very frequent DNA mutations and impairment of apoptosis lead to the development of highly anaplastic, therapy-resistant, neoplastic cells that are capable of causing local tumour recurrence and/or distant metastases. Moreover, cancer cells develop effective mechanisms that can help them to avoid the toxic effects of free radicals, although the amount of ROS tolerated by neoplastic cells is limited and above certain levels of oxidative stress or during prolonged exposure, the self-defence mechanisms become overwhelmed and cell death occurs [2]. Administration of HBO can be therapeutically advantageous by providing additional oxygen molecules and thereby increasing chemotherapy-generated oxidative stress. In addition, the transport of cytostatic agents is hindered by poor tissue perfusion [2, 45]. HBO treatment overcomes tissue hypoxia and promotes the development of new blood vessels, which are then able to transport drug molecules, thereby rendering the tumour cells more sensitive to chemotherapeutic treatments. Several studies have been conducted to examine the impact of hyperbaric oxygen as an adjuvant agent for use with chemotherapy [46–60]. Kalns et al. [47] found that the effect of hyperbaric oxygen on the proliferation and sensitivity to chemotherapy of prostate cancer cells in vitro were dependent on the type of cell line. Alazog et al. [46] examined the effect of cisplatin treatment on ovarian cancer in mice xenografts under HBO administration and found that HBO improved tumour vascularization and, when combined with cisplatin, diminished the tumour growth more effectively than cisplatin alone. Similar results were also obtained by Selvendiran et al. [58]; however, the authors believed that the remarkable lessening of the tumour volume was mostly associated with a dramatic reduction in overall body weight in the cisplatin–HBO group. The probable cause was an extreme increase in cisplatin toxicity triggered by hyperbaric oxygen, which led the authors to conclude that the combination of cisplatin and HBO should be avoided. In a study performed on sarcoma cells in vivo, Takiguchi et al. [48] assessed the therapeutic impact of HBO alone as well as in combination with 5-fluorouracil (5-FU). They found only a slight difference in tumour size between the control and the HBO monotherapy group. In contrast, the combination of 5-FU and HBO together significantly slowed the progression of tumour growth in comparison with the both the control group and the group treated with administration of 5-FU alone. Moreover, Stuhr et al. [50] observed that the combination of 5-FU and HBO can cause regression of mammary tumours. Moen et al. evaluated the influence of HBO on the uptake of 5-fluorouracil in an in vivo model of mammary adenocarcinoma in rats. Three groups were studied: a control group under atmospheric conditions (defined by the authors as 1 bar), a group subjected to a single HBO treatment (pO_2_ = 2 bar), and a group subjected to repeated HBO treatments (pO_2_ = 2 bar, single treatment every third day of experiment, four series in total) [56]. Administration of a single dose of HBO caused 5-FU uptake into the tumour tissue to rise, as measured immediately after oxygenation. No differences were observed between the control group and the group with the repeated HBO treatments, although it is noted that the measurements of 5-FU uptake of the third group were taken 24 h after the last HBO session, which may be too long a time lag to observe any effects, as the pO_2_ increase in tissue lasted for only about 60 min. Ohguri et al. [60] proposed to combine hyperbaric oxygen with carboplatin and mild hyperthermia (HT). After using carboplatin–mild HT–HBO treatment sequence, the tumour growth was slowed down and its size was reduced. It may be concluded that HBO might be a promising chemotherapy adjuvant, although its observed effectiveness depends strongly upon the cytotoxic agent and the experimental conditions under which is it measured, as well as the type of the tumour.

## Hyperbaric oxygen therapy in malignant glioma treatment

Gliomas are the most common primary brain tumours, derived from astroglial cells. The highly malignant tumour, glioblastoma (GBM), is characterized by aggressive biology and infiltrative growth. The standard treatment for glioblastoma includes surgical intervention, radiotherapy, and chemotherapy. Despite continuous advancements in treatment strategies, the prognosis for malignant glioma patients remains poor. Median survival after GBM diagnosis is about 12 months and <15 % of patients live more than 2 years [61]. Even the administration of temozolomide (TMZ), the chemotherapeutic agent most commonly used for gliomas, extends expected survival by only 2 months. It is thought that one of the pivotal factors responsible for treatment failure is tumour hypoxia, which is a state of reduced oxygen availability, or decreased oxygen partial pressure, that restricts or even abolishes cell functions [62]. Neoplastic proliferation induces rapid tumour growth, associated with dysregulated and faulty angiogenesis, resulting in a condition in which the neoplastic cells that are located in deeper regions of the tumour are poorly supplied with oxygen [62, 63]. Such cells migrate along the pathway of white matter to reach the better oxygenated brain areas proximal to blood vessels. Moreover, the hypoxia itself activates mechanisms responsible for formation of abnormal blood vessels. Therefore, invasion of neoplastic cells into the normal brain tissue and faulty angiogenesis establish two so-called vicious circles, in which a major causative role can be ascribed to the occurrence of local hypoxic regions within the tumour mass [63, 64]. Whereas hypoxia is toxic to normal cells, causing their apoptotic and necrotic death, neoplastic cells acquire the ability to develop defence mechanisms that allow them to survive under conditions of oxygen deprivation [2, 64]. The partial pressure of oxygen differs within normal tissues and solid tumours and is between 24 and 66 mmHg and 2.5–30 mmHg, respectively [65]. Hypoxia triggers changes in the expression of proteins involved in the cell cycle, proliferation, metabolism, and apoptosis, which can exacerbate tumour malignancy [2]. Consequently, a high level of hypoxia is considered to be a negative prognostic factor with respect to patient survival and response to treatment. Poor vascularization and insufficient perfusion inside the tumour mass limit the action of cytostatic agents, as they reach distant tumour cells in lower concentrations than could otherwise be expected. The administration of higher doses of cytostatic drugs is not an alternative, due to their toxicity towards normal, healthy cells [2]. Oxygen deprivation also results in decreased efficiency of radiotherapy and phototherapy [62, 66]. Therefore, to potentiate treatment, various strategies to overcome tumour hypoxia have been proposed, including inhibition of the expression of hypoxia inducible factors (HIFs), increasing the ability of red blood cells (RBCs) to carry oxygen (transfusions, administration of erythropoietin), and the use of artificial oxygen carriers (haemoglobin- or perfluorocarbon-based), radiosensitizers, bioreductive drugs, and gene therapy [65–68]. One of the most promising approaches to address the problem of tumour hypoxia appears to be HBOT.

Only a few reviews addressing the use of HBOT in patients with brain tumours have been published to date [69]. Kohshi et al. [70] focused on the therapeutic effects of HBO on brain tumours and tissue that has been injured by radiation. In normal brain tissue, oxygen is observed at approximately 25–50 mmHg [71, 72]. It is well documented that inside brain tumours, as in other solid tumours, oxygen transport is seriously hindered and that local hypoxic regions develop. As has been mentioned previously, the application of HBO increases oxygen transport *via* blood plasma, independently from transport *via* haemoglobin. It has been experimentally demonstrated that inhibition of oxygen saturation of haemoglobin to a level of 75 % induced the reduction in partial pressure of oxygen in the cerebral tissue of rats that were breathing air, whereas no pO_2_ changes were detected in rats that were breathing high-pressure oxygen [73]. A positive impact of HBO on the oxygenation of brain tissue can be observed both in the normal brain as well as in glioblastoma tissue; the oxygen level can rise by as much as 100–115 % upon HBO exposure [74]. Beppu et al. [75] measured peritumoral and intratumoral pO_2_ in patients with glioblastoma under different conditions. The mean intratumoral pO_2_ level was 9.2 ± 5.8 mmHg, which is consistent with the findings obtained in earlier studies [76, 77]. Hyperbaric oxygenation caused an elevation in pO_2_ levels in the tissue most closely surrounding the tumour and in the tumour interior, and in both of these the pO_2_ level remained greater than 30 mmHg for 15 min after decompression [75].

Stuhr et al. [78] elucidated the effects of hyperbaric oxygen alone on BT4C rat glioma xenografts in vivo. Groups of eight animals were treated with 100 % O_2_ under normobaric conditions or under higher pressure (of 2 bar), or kept under normal air conditions as the control. Hyperoxia (both normo- and hyperbaric) resulted in a 60 % retardation of tumour growth in comparison with tumour growth in the controls. Additionally, such tumours showed a greater number of “empty” vacuole spaces and necrotic areas, with 20 % more apoptotic cells than were observed in control tumours, although it was notable that the proliferation rate of cells remained unchanged. Under both hyperoxia conditions, gene profile analysis revealed induction of several pro-apoptotic genes (*GzmG*, *Grid1*, *Ceacam1*) and proliferation inhibitors (*Gap1gal*), as well as reduction in the expression of several anti-apoptotic (*Accn1*, *Nup62*, *Psen1*), pro-proliferation (*Hyal4*, *Sn5atp9*), and pro-angiogenic genes (*Hif2α*, *Efna1*). HBO did not cause damage of normal tissues. Inconsistent observations were made by Wang et al. [79]. Administration of HBO to intracranial transplanted glioma mouse model caused intensification of tumour growth as well as glioma cells proliferation and angiogenesis. However, apoptosis of neoplastic cells was significantly higher in HBO group than in control. Ding et al. also pointed out to the potentially hazardous consequences of HBO treatment like increased tumour volume and angiogenesis, likewise apoptosis inhibition [80]. Nevertheless, once again HBO procedures employed to the mentioned studies were more intensive than previously used. Such discrepancy might result also from using the intracranial glioma models, while so far experiments were carried out on subcutaneous transplanted glioma cells.

Independently of uncertain beneficial effect of HBO alone, its combination with radio- and/or chemotherapy appeared advantageous. Chang investigated whether the application of HBO during irradiation might improve the curative effect of radiotherapy on glioma treatment [81]. The median survival time of the patients in the HBO group was 38 weeks, in comparison with 31 weeks for patients in the control group, and survival rates after 18 months were 28 and 10 %, respectively. Dowling et al. conducted a pilot study to investigate the hypothesis that a combination of perfluorochemicals with hyperbaric oxygen as adjunct for radiation may have more beneficial impact than either adjuvant therapy alone [82]. Sixteen of the 20 patients with anaplastic astrocytoma or glioblastoma, who received Fluosol prior to irradiation in a HBO atmosphere, completed the medical treatment with no symptoms of oxygen toxicity. Despite enthusiasm generated by the outcomes of the trials reviewed above, the effect of HBO simultaneously applied with radiotherapy turned out to be complicated, as it resulted in late side effects, including radiation necrosis and seizures [83]. Kohshi et al. [83] proposed a variant approach, in which patients were irradiated immediately after decompression from the HBO conditions. Although reduction in high-grade gliomas was observed in 33 % of the patients in the control irradiated group, they all suffered recurrences and died within 36 months after radiotherapy. In the group treated with HBO and RT, more than 50 % tumour regression was observed, and in four of the nine patients with anaplastic astrocytoma the disappearance of the tumour lesions was demonstrated by neuroimaging. Three patients from the HBO-treated group died due to neoplastic development during the period of observation. Kohshi et al. [84] further investigated the optimal time window during which external beam radiotherapy should be applied after hyperbaric oxygenation to achieve the desired therapeutic effect of treatment of patients with malignant gliomas. The median survival was observed to be twice as long in the hyperbaric groups than in the control group. Greater than 50 % tumour regression was observed in patients who received RT 15 min after decompression. In the group receiving radiotherapy 30 min after decompression, no cancer regression occurred, but the tumours were stable during an 11–14 month follow-up period. The reason was postulated to be that the higher oxygen pressure in the tumour lasted only for a short time after oxygenation. Kohshi et al. [85] also examined the effect of combining HBO with fractionated stereotactic radiotherapy (FSRT) using a gamma unit. FSRT allows the precise, and therefore less invasive, administration of lower doses of radiation, which reduces damage to the normal tissue in the vicinity of tumour. The median survival time of patients treated with this protocol was observed to be 19 and 11 months for patients with anaplastic astrocytoma and glioblastoma, respectively. Bühler et al. [86] suggested that photon irradiation applied after HBO can extenuate glioblastoma cells survival and migration. Analysis of prospective studies on the influence of HBO followed by RT on high-grade glioma patients showed that proposed treatment is safe and gives promising therapeutic results [87].

In Japan, malignant glioma is most commonly treated with interferon beta, nimustine (ACNU), and radiation (IAR therapy). Beppu et al. [88] tested a modified IAR therapy protocol in which radiation was preceded by the administration of hyperbaric oxygen. A response to HBO/IAR treatment was observed in 10 (50 %) of the patients with glioblastoma and 3 (30 %) of the patients with anaplastic astrocytoma (43 % in total). Only in two cases was tumour progression noted. No correlation was made between response rates and other prognostic factors including age, Karnofsky performance scale (KPS), histological type of tumour, tumour size, and tumour location. The results suggested that HBO/IAR may be advantageous for patients with poor prognosis, who achieved comparable results to those with good prognosis. Meanwhile, Ogawa et al. conducted a prospective trial to investigate the feasibility and efficacy of radiotherapy after hyperbaric oxygenation used together with chemotherapy [89]. Patients with various kinds of malignant gliomas, including glioblastoma, anaplastic astrocytoma, anaplastic oligoastrocytoma, and anaplastic oligodendroglioma, were exposed to radiation treatment within 15 min after HBOT. In addition, they received the cytostatic agents, procarbazine, nimustine, and vincristine. A 69 % positive response was observed, with no instance of disease progression. The one- and two-year overall survival rates were 83 and 56 %, respectively. The same team observed similar trends in further studies enlisting a larger group of patients [90]. A positive response was observed in 57 % of the cases, and the median survival time for patients with glioblastoma was slightly prolonged from 11–15 months to 17.3 months in this study. However, acute toxicity developed in almost 48 % of the patients in both trials. Nevertheless, grade 4 haematological toxicity and late radiation injuries were observed to be rare, and the use of this method is not precluded. Over the long term, the median survival time of patients treated with HBO followed by radiotherapy in combination with chemotherapy was 20.2 months, with a 17.2 and 113.4 months median observed for patients with glioblastoma and grade III gliomas, respectively [91].

To investigate the influence of HBO on the distribution of carboplatin in the brain, Suzuki et al. [92] used a rat model in which this cytostatic agent was administered intravenously. Under atmospheric conditions, they found the level of carboplatin in rat brains to be undetectable, as opposed to HBO-treated groups, in which the drug concentration could be established. The same research team evaluated the effectiveness of carboplatin combined with HBO for treatment of patients with gliomas [93]. HBO treatment resulted in a prolongation of the carboplatin mean residence time (MRT) in plasma. However, the effects observed in patients were diverse (from complete recovery or reduction of tumour mass to progression of disease) and also difficult to interpret, due to the small number of patients in each of the treatment groups. To assess the impact of HBOT on the therapeutic effect of temozolomide (TMZ), Dagistan et al. [94] conducted an experimental study on rats that had been injected with C6 glioma cells. The results of this study suggested that the combination of TMZ and HBO significantly diminishes cell proliferation and angiogenesis and that it also stimulates apoptosis of glioma cells, in comparison with the control group and the group treated with TMZ alone. The potential influence of HBO on TMZ cytotoxicity was independently examined by Lu et al. [95] in in vitro trials. The human glioma U251 cell line was incubated under various therapeutic conditions: control, HBO alone, temozolomide alone, and temozolomide plus HBO. Analysis of the outcomes showed that combined TMZ/HBO therapy provided significantly better cell growth inhibition, compared to HBO alone, which elicited no changes. HBO also induced apoptosis of neoplastic cells. Furthermore, the mean rate of apoptosis was found to be over two and four times greater in the TMZ-treated and the TMZ/HBO group, respectively, compared to controls. A similar effect was observed with regard to cell viability; the TMZ group exhibited a fourfold increase in per cent of dead cells, compared to the control group, while in the TMZ/HBO group the percentage of dead cells was increased sixfold. Decreased VEGF and MRP-1 protein levels that were observed in the HBO and TMZ/HBO experimental groups suggested that HBO may elicit its effect through two pathways: inhibition of angiogenesis and diminution of drug resistance.

## Final conclusions

Hypoxic regions within the tumour mass play a major role in tumour development and resistance to novel radio- and chemotherapies. HBOT holds promise as an approach to overcoming oxygen insufficiency by increasing the oxygen supply to neoplastic tissue. Recent results strongly indicate that HBO does not induce cancer growth, recurrence, or metastasis. Indeed, HBO is observed to exert an inhibitory effect on cancer cell proliferation and to stimulate cancer cell apoptosis. However, the beneficial effect of HBO is diverse, and it varies with the tumour type, malignancy, size of the lesion, and the clinical state of the patient. It is dependent on the specifics of the oxygenation protocol, and consequently it is very important to establish the proper moment of application, duration, pressure, and number of doses.

We conclude that the administration of HBO can provide many clinical benefits in the treatment of tumours, including management of highly malignant gliomas. Applied immediately before irradiation, it is safe and well tolerated by patients, causing rare and limited side effects. The results obtained with a combination of HBO/radiotherapy protocol proved to be especially favourable compared to radiation treatment alone. HBO can also increase the cytostatic effect of certain drugs, which may render standard chemotherapy more effective. The currently available data support the legitimacy of conducting further research on the use of HBO in the treatment of malignancies.
